# The impact of novel coronavirus (2019-*n*CoV) pandemic movement control order (MCO) on dengue cases in Peninsular Malaysia

**DOI:** 10.1016/j.onehlt.2021.100222

**Published:** 2021-01-29

**Authors:** Mohd Hafiz Rahim, Nazri Che Dom, Sharifah Norkhadijah Syed Ismail, Zamzaliza Abd Mulud, Samsuri Abdullah, Biswajeet Pradhan

**Affiliations:** a Centre of Environmental Health & Safety, Faculty of Health Sciences, UITM Cawangan Selangor, Universiti Teknologi MARA (UiTM), 42300 Puncak Alam, Selangor, Malaysia; b Integrated Mosquito Research Group (I-MeRGe), UITM Cawangan Selangor, Universiti Teknologi MARA (UiTM), 42300 Puncak Alam, Selangor, Malaysia; c Institute for Biodiversity and Sustainable Development (IBSD), Universiti Teknologi MARA, 40450 Shah Alam, Selangor, Malaysia; d Department of Occupational Health and Safety, Faculty of Medicine and Health Sciences, Universiti Putra Malaysia, 43400 Serdang, Selangor, Malaysia.; e Faculty of Health Sciences, Centre of Nursing Studies, UITM Cawangan Selangor, Universiti Teknologi MARA (UiTM), 42300 Puncak Alam, Selangor, Malaysia; f Faculty of Ocean Engineering Technology and Informatics, Universiti Malaysia Terengganu, 21030 Kuala Nerus, Terengganu, Malaysia; g The Centre for Advanced Modelling and Geospatial Information System (CAMGIS), Faculty of Engineering and Information Technology, University of Technology Sydney, Sydney 2007, NSW, Australia; h Department of Energy and Mineral Sources Engineering, Sejong University, Choongmu-gwan, 209, Neungdong-ro, Gwangin-gu, Seoul 05006, Korea; i Center of Excellence for Climate Change Research, King Abdul Aziz University, P.O.Box 80234, Jeddah 21589, Saudi Arabia

**Keywords:** MCO, Dengue cases, COVID-19, GIS, Malaysia

## Abstract

This study has highlighted the trend of recently-reported dengue cases after the implementation of the Movement Control Orders (MCOs) caused due to COVID-19 pandemic in Malaysia. The researchers used the dengue surveillance data published by the Malaysian Ministry of Health during the 3 phases of MCO (which ranged between 17th March 2020 and 28^th^ April 2020) was used for determining the cumulative number of dengue patients. Thereafter, the dengue cases were mapped using the Geographical Information System (GIS). The results indicated that during the 42 days of MCO in Peninsular Malaysia, 11,242 total cases of dengue were reported. The daily trend of the dengue cases showed a decrease from 7268 cases that occurred before the MCOs to 4662 dengue cases that occurred during the initial 14 days of the COVID-19 pandemic (i.e., MCO I), to 3075 cases occurring during the MCO II and 3505 dengue cases noted during MCO III. The central peninsular region showed a maximal decrease in new dengue cases (52.62%), followed by the northern peninsular region (1.89%); eastern coastal region (1.25%) and the southern peninsular region (1.14%) during the initial MCO implementation. However, an increase in the new dengue cases was noted during the MCO III period, wherein all states showed an increase in the new dengue cases as compared during MCO II. The decrease in the pattern was not solely based on the MCO, hence, further investigation is necessary after considering different influencing factors. These results have important implication for future large-scale risk assessment, planning and hazard mitigation on dengue management.

## Highlights

• Dengue has been described as a “silent killer” amidst the COVID-19 pandemic.
• The MCOs implemented by the Malaysian government to break the COVID-19 infection chain have positively affected the onset of new dengue cases.
• The highest reduction of new dengue cases were reported in the central peninsular region (52.62%), followed by the northern peninsular region (1.89%), eastern coastal region (1.25%) and the southern peninsular region (1.14%).
• New dengue cases increased during the MCO3 period, with the increase of reported dengue cases in all states in Peninsular Malaysia.

## 1 Introduction

The coronavirus disease, 2019 (COVID-19) originates from a novel CoV, which has a 79% genome sequence similarity, with the coronavirus that triggers the severe acute respiratory syndrome (SARS-CoV). Initially known as 2019 novel coronavirus (2019-nCoV) [12], it was eventually renamed SARS-CoV-2 [16]. The SARS-CoV-2 epidemic flared up in Wuhan, in the Hubei Province of the People’s Republic of China, towards the close of 2019 [13]. In response to the global threat posed by this outbreak, the World Health Organization (WHO) proclaimed COVID-19 a Public Health Emergency of International Concern (PHEIC) [26]. From the People’s Republic of China, the tentacles of COVID-19 soon made their way to other Asian countries (including Japan, Thailand, Singapore and Malaysia), Australia, Europe and North America [23]. Initially detected on the 25^th^ of January, 2020 [20], the number of COVID-19 infections in Malaysia began to rise relentlessly, particularly during the month of March, 2020. This alarming disclosure led to the Malaysian government’s introduction of several countermeasures. These included the installing of an inspection system to quickly diagnose infected individuals, the carrying out of instantaneous case isolation and thorough tracking, and the quarantining of those verified to have been in close contact with individuals diagnosed positive. Also, in order to isolate the sources of COVID-19 infections, the movement control order (MCO) was implemented nationwide. According to available data, at the close of Phase I of the MCO (31^st^ March, 2020), 2766 COVID-19 positive cases were recorded, while at the conclusion of Phase II (14^th^ April, 2020), 4987 positive cases were recorded [20]. While essential services were given the green light to operate during the MCO, many other activities, including business operations, were put on hold [18].

This MCO was based on the Police Act 1967 and the Prevention and Control of Infectious Disease Act (PCID Act 1988) [20]. The MCO I was implemented in the country between 18^th^ and 30^th^ March 2020. This MCO was primarily implemented for controlling and decreasing the spread of COVID-19 in Malaysia, since a sudden spike was noted in the reported cases due to several Islamic religious gatherings, consisting of 12,000 people, which took place in Sri Petaling, Kuala Lumpur, between 28^th^ February and 2^nd^ March 2020. The Malaysian Prime Minister declared the MCO I on 16^th^ March 2020. This was gazetted in the [30] (PCID Order) on 17^th^ March 2020, and all the 14 Malaysian states were declared to be locally-infected regions. For guiding this implemented order and managing the MCO, the government also issued a regulation according to the [30] (PCID Regulations), which highlighted the need to - (i) Control the movement and gathering of people in the infected areas; (ii) Make it mandatory to examine the health status of every individual arriving in the country; (iii) Restrict the operating hours of essential services; and (iv) Stipulate penalties for the non-compliance of these PCID regulations. This Malaysian MCO was based on a similar initiative implemented by other countries like China, UK, India, Italy, Spain, France, etc., which restricted the movement of people. Following the implementation of the MCO included a few vital courses of action; 1) a full movement prohibition and broad national meetings, 2) Full restriction of all Malaysian travel abroad; 14 days quarantine is required for returnees abroad, 3) Full limitation of the entry to the country for both visitors and foreigners. 4) Children's, kindergartens, public and private school closures. 5) Closure of all institutions of higher education and training centres. 6) The closure, except for those offering critical services, of all government and private buildings including health [9]. The police, armed forces, civil defense force and the paramilitary civil volunteer corps were deployed as front liners to enforce the MCO, and to assist with the supply logistics of medicines and personal protective equipment.

Vector control and management, community participation, and enforcement are the main components, of the three-pronged dengue control strategy employed by the Malaysian government. Vector control mostly entails the extensive use of insecticides, to diminish the mosquito population, in areas where positive cases have been detected. The space spray application of insecticides (particularly pyrethroids) is executed in the cold or thermal fogging mode (ref.). Surveillance mostly involves house-to-house inspections to detect mosquito larvae infestations, with the results applied for the formulation of indicators that include the house index and the Breteau index.

Currently, with the focus of government officials mainly on curbing the spread of the COVID-19 virus, the regular operations of the dengue control system have been significantly curtailed. This situation has led to a worrying climb in the number of dengue cases among Malaysians. In 2018, Malaysia recorded 80,615 dengue cases with 147 fatalities, while in 2019, 130,101 cases were recorded with 182 fatalities. According to the CPRC’s Dengue Operations Centre (http://idengue.arsm.gov.my/), between the 29^th^ of December, 2019, and the 22^nd^ of April, 2020, Malaysia recorded a total of 38,464 dengue cases, and a death toll of sixty-three individuals. Topping the list of Malaysian states during this period, in terms of dengue cases, was Selangor with 23,140 cases, followed by Johore with 3,181 cases, Kuala Lumpur with 2,932 cases, Sabah with 2056 cases, Kelantan with 1,441cases and Perak with 1,255 cases. It is notable that following the enforcement of the MCO, the work from home trend served to hinder the movement of the dengue-transmitting host.

This investigation seeks to identify variation in the trend and pattern of the dengue cases, prior and subsequent to the enforcement of the MCO in Peninsular Malaysia. It is our contention, that the results derived through this investigation, will serve to make clear the impact of the COVID-19 outbreak, and the consequential implementation of the MCO, on Malaysia’s dengue situation.

## 2 Methods

**Fig. 1:**
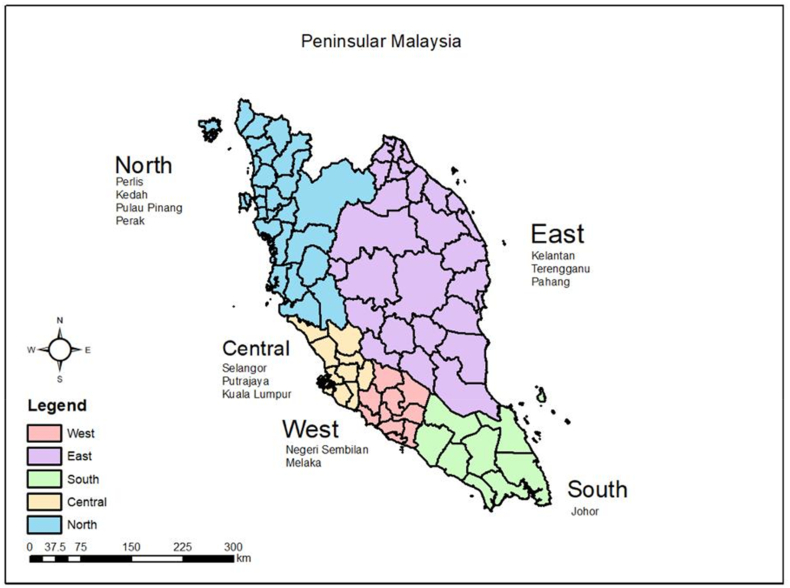
The visualization of the 91 districts in Peninsular Malaysia. For reporting purposes, this map was divided into five main regions namely (i) Northern Region (Perlis, Kedah, Penang, Perak), (ii) East Coast Region (Kelantan, Terengganu, Pahang), (iii) Central Region (Selangor, Kuala Lumpur, Putrajaya) (iv) West region (Negeri Sembilan & Malacca) and (v) Southern Region (i.e. Johor).

Malaysia lies in Southeast Asia and occupied some regions of the Malay Peninsula and Borneo Island. Peninsular Malaysia shows the total size of 132,265 km^2^, and occupies 40% of the total country’s area. It includes 11 states and 2 federal territories. This region is divided into 5 regions, i.e., Northern Region (which includes Perlis, Kedah, Penang, Perak), Eastern-Coast Region (which includes Kelantan, Terengganu, Pahang), Central Region (that includes Selangor, Kuala Lumpur, Putrajaya) and the West Region (that includes Negeri Sembilan, Malacca), (Southern Region (Johor). This study focuses on the 5 major regions in Peninsular Malaysia. Malaysia has a total population of 32.73 million [4], wherein the Central region shows the highest population, followed by the Southern and the Easter-Coast regions (Fig. 1).

All data for the dengue cases for different regions were collected from the Crisis Preparedness and Responses Centre (CPRC), [3] and Dengue Operations Centre website (http://idengue.arsm.gov.my/). This data was collected for the period ranging between 2^nd^ March and 12^th^ May 2020, for understanding the changing trend (%) in the dengue cases. The Malaysian government implemented the MCO in 4 phases, i.e., Phase I (MCO I -from 18^th^ to 31^st^ March 2020); Phase II (MCO II - from 1^st^ to 14^th^ April 2020); Phase III (MCO III -from 15^th^ to 28^th^ April 2020) and the Phase IV (MCO IV from 29^th^ April to 12^th^ May 2020). In this study, the data from the initial 3 MCO phases were included. The data consisted of the number of reported dengue cases for every district in Peninsular Malaysia. Furthermore, the data was based on the epidemiological weeks related to the dengue cases before and during the MCO phases, i.e., MCO I (Epid week 12 - 13), MCO II (Epid week 14 -15) and MCO III (Epid week 16 - 17). This data covered different Health Offices in Peninsular Malaysia that were categorised into 4 regions, i.e., Northern region (*n* = 28 districts), Central region (*n* = 14 districts), Southern region (*n* = 20 districts) and Eastern Coastal Region (*n* = 29 districts). The data for 91 districts in Peninsular Malaysia were included in this study and are described in Table 1.

**Table 1:** Dengue cases by regional and district in Peninsular Malaysia.

Region	State	District Health Office
North	Perlis	Kangar
	Kedah	Baling, Bandar Baharu, Kota Setar, Kuala Muda, Kubang Pasu, Kulim, Langkawi, Padang Terap, Sik, Yan, Pendang
	Penang	Barat Daya, Seberang Perai Utara, Seberang Perai Selatan, Seberang Perai Tengah, Timur Laut
	Perak	Batang Padang, Kinta, Kampar, Hulu Perak, Manjung, Selama, Hilir Perak, Perak Tengah, Muallim, Kuala Kangsar, Kerian
Central	Putrajaya	Putrajaya
	Kuala Lumpur	Cheras, Kepong, Titiwangsa, Lembah Pantai
	Selangor	Petaling, Klang, Gombak, Hulu Langat, Sepang, Hulu Selangor, Kuala Selangor, Kuala Langat, Sabak Bernam
West	Negeri Sembilan	Seremban, Port Dickson, Jempol, Kuala Pilah Tampin, Rembau, Jelebu
	Malacca	Melaka Tengah, Alor Gajah, Jasin
South	Johor	Batu Pahat, Johor Bharu, Kluang, Kota Tinggi, Kulai, Mersing, Muar, Pontian, Segamat, Tangkak
East	Pahang	Bentong, Bera, C.Highlands, Jerantut, Kuantan, Lipis, Maran, Pekan, Raub, Rompin, Temerloh
	Terengganu	Besut, Dungun Kemaman, Kuala Nerus, Kuala Terengganu, Marang, Setiu, Hulu TerengganU
	Kelantan	Bachok, Gua Musang, Jeli, Kota Bharu, Kuala Krai, Machang, Pasir Mas, Pasir Puteh, Tanah Merah, Tumpat

Note: The sites are described as DO = District Health Office in Peninsular Malaysia. It was divided into five regions

First, the variation in the dengue cases was determined based on a percentage decrease in the cases between the different periods, i.e., before the implementation of MCO and MCO I; MCO I and MCO II; and the MCO II and MCO III. This decrease in the number of dengue cases has been tabulated and mapped for understanding the varying trend in every region 2 weeks before the implementation of the MCO I until MCO III. Next, the distribution pattern of the dengue cases for every state was mapped using the GIS software.

## 3 Results and discussion

**Fig. 2:**
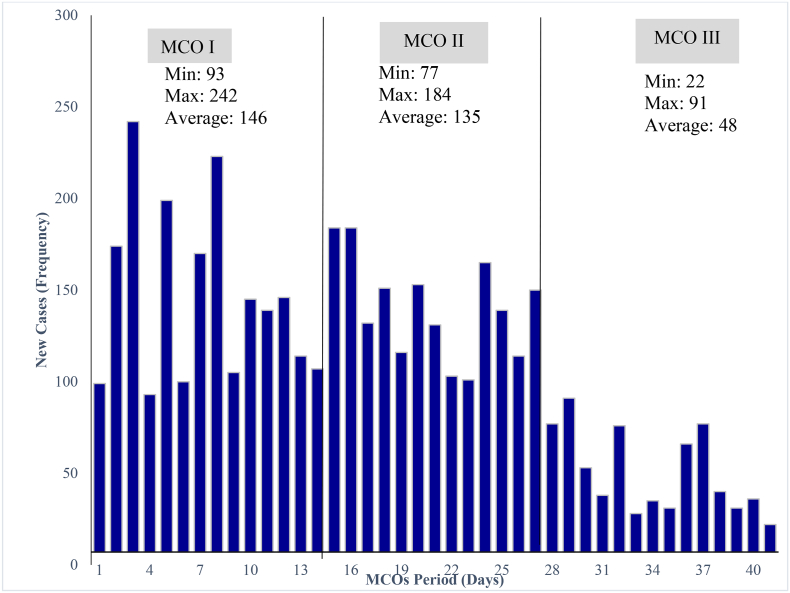
Daily COVID-19 cases reported during implementation of early MCO.

The implementation of MCO shows a significant reduction in the number of new COVID-19 cases (Fig. 2). During the first 14 days of epidemics (MCO I), 1,949 cases of COVID-19 were reported (incidence rate (IR) of 7.54 per 100,000 population), followed by 1,930 cases of COVID-19 in MCO II (IR 7.47 per 100,000 population) and 701 cases during MCO III (IR 2.71 per 100,000 population). In average, the daily trend of COVID-19 case shows a decrease pattern from 146 cases that recorded in MCO I to 135 cases that occurred during the initial 14 days of COVID-19 pandemic (MCO II), to 48 occurring during the MCO III.

The MCOs, which were implemented by the Malaysian government for breaking the COVID-19 infection chain, have also impact on the onset of new dengue cases. A comparison of the number of new dengue cases before and after the enforcement of MCOs indicated that there has been a significant decrease in the dengue cases. The initial number of dengue cases before and after MCO implementation ranged from 3 to 5804 cases and 0 to 2336 cases, respectively. Out of all the regions, the Central Peninsular region showed the highest decrease in the number of dengue cases, from 6164 to 2340 cases (i.e., 62.03%) followed by the Northern region (i.e., from 257 to 120 cases, i.e., 53.30%); Eastern coastal region (from 244 to 154 cases; i.e., 35.43%), West coast region (from 233 to 174 cases; i.e., 25.32%) and Southern region (from 370 to 287 cases; i.e., 22.43%). According to the Control and Prevention of Vector Borne Disease Program, the Malaysian Ministry of Health defined a dengue outbreak as an occurrence of ≥ 1 dengue cases in the locality or an occurrence of dengue case during the incubation period of the initial case or the index case which was notified to the relevant authorities. The District Health Office used this definition for implementing immediate control measures. Before the MCO, the Petaling District Health Office in Selangor reported the highest number of dengue cases, i.e., 1841 cases. Table 2 presents the difference in the cumulative number of the dengue cases before and during the implementation of the MCO.

**Table 2:** Variation of daily dengue cases 2 weeks before MCO and during MCOs enforcement.

Region	State	District	Before MCO	MCO I	MCO II	MCO III	Variation of dengue cases		
							MCO I (%)	MCO II (%)	MCO III (%)
Northern	Perlis	Kangar	3	0	1	0	−0.04%	0.02%	-0.03%
	Kedah	Baling	10	27	6	7	0.23%	−0.45%	0.03%
		Bandar Baharu	1	1	0	0	0.00%	−0.02%	0.00%
		Kota Setar	7	3	3	1	−0.06%	0.00%	−0.07%
		Kuala Muda	21	3	4	3	−0.25%	0.02%	−0.03%
		Kubang Pasu	6	2	2	1	−0.06%	0.00%	−0.03%
		Kulim	12	2	6	1	−0.14%	0.09%	−0.16%
		Langkawi	0	0	0	0	0.00%	0.00%	0.00%
		Padang Terap	0	0	0	0	0.00%	0.00%	0.00%
		Sik	1	1	0	1	0.00%	−0.02%	0.03%
		Yan	1	0	0	0	−0.01%	0.00%	0.00%
		Pendang	2	4	0	3	0.03%	−0.09%	0.10%
	P.Pinang	Barat Daya	5	5	0	0	0.00%	−0.11%	0.00%
		Seberang Perai Utara	5	11	2	2	0.08%	−0.19%	0.00%
		Seberang Perai Selatan	3	0	0	0	−0.04%	0.00%	0.00%
		Seberang Perai Tengah	6	3	4	3	−0.04%	0.02%	−0.03%
		Timur Laut	19	7	4	5	−0.17%	−0.06%	0.03%
	Perak	Batang Padang	15	6	14	15	−0.12%	0.17%	0.03%
		Kinta	102	61	55	98	−0.56%	−0.13%	1.40%
		Kampar	10	2	8	2	−0.11%	0.13%	−0.20%
		Hulu Perak	2	2	2	1	0.00%	0.00%	−0.03%
		Manjung	8	2	4	6	0.11%	0.04%	0.07%
		Lm&Selama	5	6	1	13	0.01%	−0.11%	0.39%
		Hilir Perak	6	8	1	2	0.03%	−0.15%	0.03%
		Perak Tengah	0	1	1	0	0.01%	0.00%	−0.03%
		Muallim	5	3	0	1	−0.03%	−0.06%	0.03%
		Kuala Kangsar	2	1	2	0	−0.01%	0.02%	−0.07%
		Kerian	0	3	0	0	0.04%	−0.06%	0.00%
Central	Putrajaya	Putrajaya	28	54	16	22	0.36%	−0.81%	0.20%
	Kuala Lumpur	Cheras	70	54	44	60	−0.22%	−0.21%	0.52%
		Kepong	69	41	25	40	−0.39%	−0.34%	0.49%
		Titiwangsa	106	109	104	141	0.04%	−0.11%	1.20%
		Lembah Pantai	87	70	44	86	−0.23%	−0.55%	1.37%
	Selangor	Petaling	1841	1132	613	832	−9.76%	−11.05%	7.12%
		Klang	1258	788	367	396	−6.47%	−8.97%	0.94%
		Gombak	823	530	361	341	−4.03%	−3.60%	−0.65%
		Hulu Langat	938	528	353	400	−5.64%	−3.73%	1.53%
		Sepang	362	180	153	124	−2.50%	−0.57%	−0.94%
		Hulu Selangor	193	144	97	101	−0.67%	−1.00%	1.53%
		Kuala Selangor	165	109	54	37	−0.77%	−1.17%	−0.55%
		Kuala Langat	175	113	90	95	−0.85%	1.15%	0.16%
		Sabak Bernam	49	36	19	10	−0.18%	−0.36%	−0.29%
West	Negeri Sembilan	Seremban	107	94	84	147	−0.18%	−0.21%	2.05%
		Port Dickson	7	8	5	6	0.01%	−0.06%	0.03%
		Jempol	7	8	5	6	0.01%	−0.06%	0.03%
		Kuala Pilah	7	0	4	4	−0.10%	0.09%	0.00%
		Tampin	3	1	2	1	−0.03%	0.02%	−0.03%
		Rembau	9	2	3	7	−0.10%	0.02%	0.13%
		Jelebu	0	1	1	0	0.01%	0.00%	−0.03%
	Melaka	Melaka Tengah	63	48	55	53	−0.21%	0.15%	−0.07%
		Alor Gajah	19	14	9	7	−0.07%	−0.11%	−0.07%
		Jasin	11	8	6	7	−0.04%	−0.04%	0.03%
South	Johor	Batu Pahat	9	8	5	2	−0.01%	−0.06%	−0.10%
		Johor Bharu	296	226	254	217	−0.96%	0.60%	−1.20%
		Kluang	7	8	4	16	0.01%	−0.09%	0.39%
		Kota Tinggi	6	2	2	2	−0.06%	0.00%	0.00%
		Pontian	8	3	2	2	−0.07%	−0.02%	0.00%
		Segamat	14	14	4	17	0.00%	−0.21%	0.42%
		Tangkak	5	3	2	2	−0.06%	−0.02%	0.00%
		Kulai	6	13	8	6	−0.001	−0.10%	−0.07%
		Mersing	8	7	4	10	−0.01%	−0.06%	0.20%
		Muar	9	3	2	1	−0.08%	−0.02%	−0.03%
East	Pahang	Bentong	2	1	0	0	−0.01%	−0.02%	0.00%
		Bera	0	1	0	2	0.01%	−0.02%	0.07%
		C.Highlands	0	0	0	0	0.00%	0.00%	0.00%
		Jerantut	3	2	1	4	−0.01%	−0.02%	0.10%
		Kuantan	91	66	67	51	−0.34%	0.02%	−0.52%
		Lipis	2	1	1	2	−0.01%	0.02%	0.03%
		Maran	3	4	1	3	0.01%	−0.06%	0.07%
		Pekan	9	1	1	2	−0.11%	0.00%	0.03%
		Raub	1	2	0	0	0.01%	−0.04%	0.00%
		Rompin	3	2	0	0	−0.01%	−0.04%	0.00%
		Temerloh	4	0	3	1	−0.06%	0.06%	−0.16%
	Terengganu	Besut	2	1	0	1	−0.01%	−0.02%	0.03%
		Dungun	0	0	0	2	0.00%	0.00%	0.07%
		Kemaman	4	1	2	1	−0.04%	0.02%	−0.03%
		Kuala Nerus	0	2	1	1	0.03%	−0.02%	0.00%
		Kuala Terengganu	3	0	1	4	−0.04%	0.02%	0.10%
		Marang	0	2	1	0	0.03%	−0.02%	−0.03%
		Setiu	0	1	0	1	0.01%	−0.02%	0.03%
		Hulu Terengganu	0	0	0	0	0.00%	0.00%	0.00%
	Kelantan	Bachok	16	15	10	9	−0.01%	−0.11%	−0.03%
		Gua Musang	1	1	1	2	0.00%	0.00%	0.03%
		Jeli	2	1	5	0	−0.01%	0.79%	−0.16%
		Kota Bharu	65	45	38	45	−0.28%	−0.15%	0.23%
		Kuala Krai	5	1	2	2	−0.06%	0.02%	0.00%
		Machang	2	2	0	0	0.00%	−0.04%	0.00%
		Pasir Mas	8	3	1	1	−0.07%	−0.04%	0.00%
		Pasir Puteh	1	1	1	1	0.00%	0.00%	0.00%
		Tanah Merah	6	4	3	0	−0.03%	−0.06%	−0.10%
		Tumpat	11	6	14	7	−0.07%	0.17%	−0.23%

A total of 4662 dengue cases have been reported during MCO I. The number of cases reported during this corresponding period is lower in comparison before MCO (from 7268 to 4662 cases; i.e., 35.85%). This decrease in the number of new dengue cases was noted across all regions in Peninsular Malaysia. The state of Selangor showed the highest decrease in the number of dengue cases (Petaling District Health Office; i.e., before MCO, they reported 1841 cases, which decreased to 1132 cases during MCO I), followed by the Klang District Health Office i.e., before = 1258 cases; during MCO I = 788 cases) and the Hulu Langat District Health Office i.e., before = 938 cases; during MCO I = 528 cases).

Table 2 presents the variations in the cumulative number of newly occurring dengue cases before MCO and MCO II. During MCO II, there were 3075 cases were reported from all regions in Peninsular Malaysia. The number of cases reported during this corresponding period is lower in comparison before MCO (from 7268 to 3075 cases; i.e., 57.69%). As compared to MCO II, it could be seen a continuous decreased in terms of new dengue cases reported during MCO I (4662 cases) and during MCO II (3075 cases), which contributed another 21.83% of total reduction of dengue cases before MCO. The variation in the cumulative number of dengue cases during MCO III are in contrary with previous MCO where, there is slight increase in the number of dengue cases was noted. Six states in Peninsular Malaysia (46.2%) showed an increasing trend in the number of newly occurring dengue cases as compared to MCO II especially in the central region. The state of Negeri Sembilan showed the highest increase in the number of dengue cases, from 104 to 171 cases (i.e., 39.18%) followed by Perak (from 88 to 138 cases; i.e., 36.23%) and Kuala Lumpur from 217 to 327 cases (i.e., 33.63%).

**Fig. 3:**
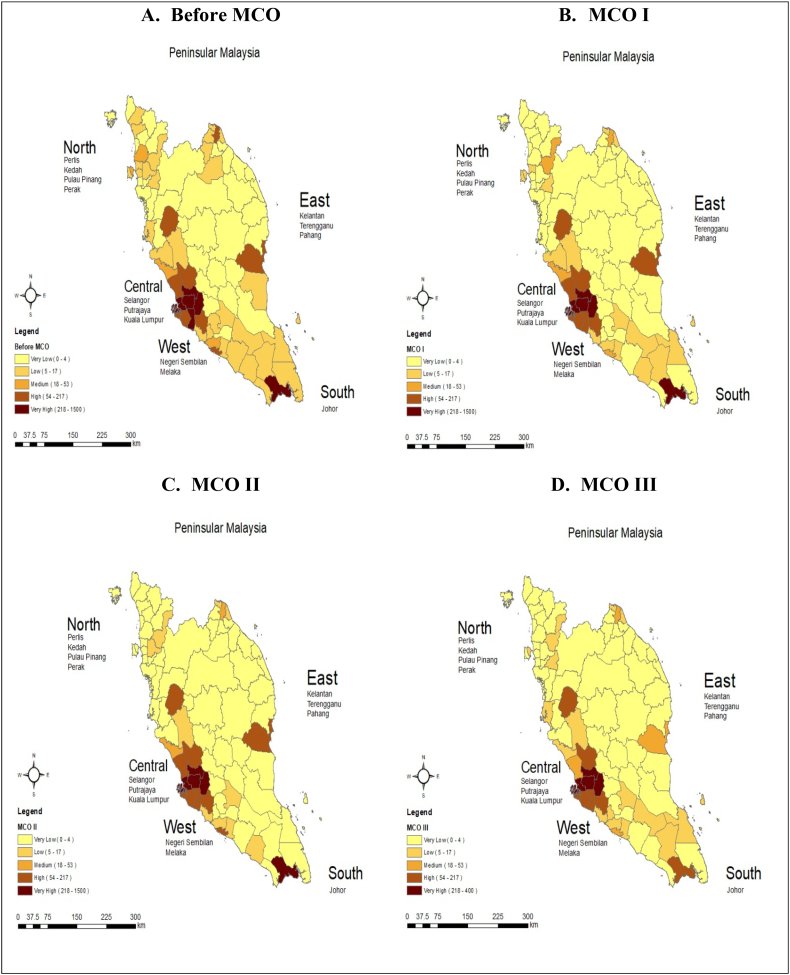
Spatial mapping of distribution pattern of dengue fever cases during MCO phases at district level. There are four phases of MCOs; Phase I (Before MCO: 2^nd^ March to 17^th^ March 2020); Phase II (MCO I: 18^th^ March to 31^st^ March 2020); Phase III (MCO II: 1^st^ April 2020 to 14^th^ April 2020), Phase IV(MCO III: 15^th^ April to 28^th^ April 2020) The data included is the number of dengue cases reported for each district in Peninsular Malaysia.

Based on the data described in the GIS-based maps, the enforcement of the MCO showed a positive effect in decreasing the frequency of the new dengue cases in the districts (Fig. 3). The decrease in the number of new dengue cases was noted across 57 districts health offices (62.6%) (Fig.3B). A significant decrease was noted in the number of dengue cases during the MCO II (Fig. 3C). The implementation of all MCOs showed a decreasing trend in the distribution of the dengue cases in a majority of the districts (68 district; i.e., 74.7%). However, an increase was noted in the period between MCO II and MCO III, specifically in the regions which already contained a higher number of cases (Fig. 3D). Furthermore, the district’s population size was seen to be the major factor, which led to an increase in the cumulative number of new dengue cases. However, the overall distribution pattern of the dengue cases in Malaysia showed a decrease from those reported in MCO I and MCO II, particularly in the Central Peninsular Region.

**Fig. 4:**
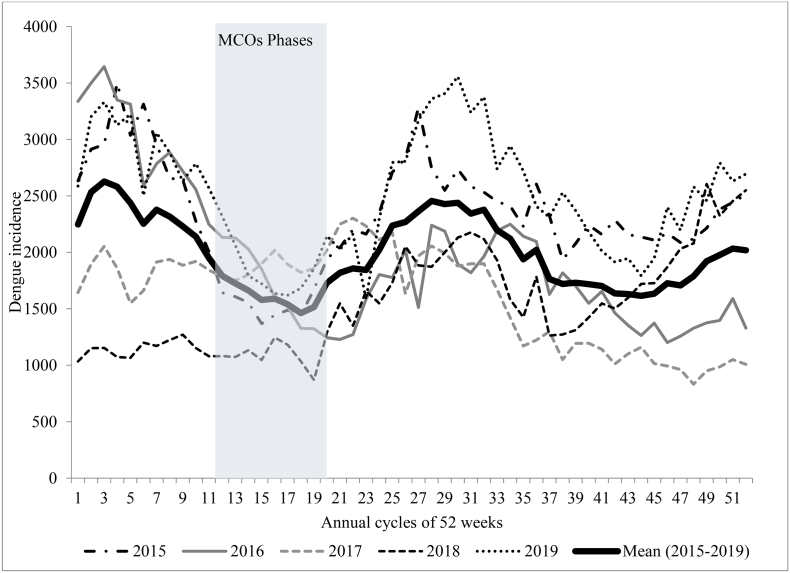
Annual cycle of dengue fever cases trend in Peninsular Malaysia from 2015 to 2019. Note: The grey area indicates the early stage of MCO implementation in Malaysia which consist of MCO I: 18^th^ March to 31^st^ March 2020, MCO II; 1^st^ April 2020 to 14^th^ April 2020, MCO III: 15^th^ April to 28^th^ April 2020).

In an effort to understand the contribution of MCO on the trends of dengue cases, this study has come out with annual cycle of 52 weeks of dengue cases by states in Peninsular Malaysia, the annual dengue fever cases from 2015 to 2019 being compared (Fig. 4). The line graph has been divided into a period of four main fraction namely before MCO, MCO I, MCO II and MCO III. Data examination showed that overall distribution of dengue fever cases during the period of MCO (8^th^ March 2020 until 28^th^ April 2020) are co-incident between the end of March to May pattern every year. Thus, indicate that the large scale of MCO of the population is not sustainable in controlling the spread of dengue.

The emergence of a previously unknown coronavirus (SARS CoV-2), which brings about the COVID-19 disease, comes with a list of clinical issues that include asymptomatic tendencies, acute pneumonia, and multi-organ problems. On March the 12^th^, 2020, the placing of the COVID-19 disease, under the category of a pandemic by the WHO, triggered the implementation of a host of preventive and containment measures all over the globe. The WHO has identified dengue and acute dengue, as the main sources of severe ailments and fatalities, among several dengue endemic countries in Asia and South America [28,29]. Already in the midst of coping with the spread of COVID-19, a surge in dengue cases would put considerable strain on the healthcare sector of these countries. Additionally, the situation is made complicated by the fact that the symptoms for dengue and COVID-19 are close to similar. The initial clinical features are somewhat similar in both dengue and COVID19, along with similar laboratory features. [15]. Gastrointestinal symptoms like diarrhoea are common in patients with acute dengue and in COVID 19, while sore throat is common with acute dengue symptoms with around 38% of patients. [6]. Therefore, it can be difficult to clinically distinguish dengue from COVID-19, particularly in countries where dengue is endemic. Only purely clinical and laboratory features may confirm this. According to Joob and Wiwanitkit [11] there is a probability that an individual with COVID-19 infection could partially have a skin rash symptom, that may be misdiagnosed as another common disease. This renders an accurate diagnosis a considerably challenging task [27].

In this study, a detailed assessment was done on the distribution of the dengue cases in Peninsular Malaysia before and during the different phases of the implementation of MCO. The MCO prohibited the mass movement and the gathering of people. These restrictions on the human movements indirectly decreased the spread of dengue virus in Malaysia, though a thorough investigation is needed for determining the effect of other factors such as environmental parameters and local meteorology. The results indicated that the MCO phases significantly decreased the number of new dengue cases since a majority of the people stayed at home, which further decreased the human movement [2]. This restricted the spread of this virus in the community and indirectly decreased the number of new dengue cases during MCO. Many studies indicated that a variation in the contact pattern could either amplify or restrict the transmission rate of the diseases. This factor also decreased the epidemic stability and the disease prevalence, and also curbed the probability of a new outbreak [1,17,19,25].

While a dip in the count of new dengue cases was observed following the implementation of MCO I, this situation took a turn for the worse, when a significant increase in cases occurred during MCO III. This increase in dengue cases can probably be put down to the cessation of vector control measures, which would have been carried out with no interruption, if the COVID-19 situation had not cropped up. During the MCO phase, the decreased emphasis on demographic surveillance, and the monitoring of infected mosquitoes, reduced the effectiveness of measures put in place to curb the dengue epidemic [14]. Thirty days into the MCO, several locations in Selangor, notably in the Petaling and Hulu Selangor districts, were proclaimed dengue hotspot areas. Public compliance to the MCO left most shops, construction sites, and public spaces virtually deserted. While this situation may serve to curb the spread of COVID-19, the lack of maintenance in these areas can lead to rainwater accumulation, which can consequently turn into breeding grounds for mosquitoes. It has been established that warm weather conditions accelerate the dengue transmission rate, while humid weather conditions promote an ideal atmosphere for the proliferation of dengue vectors.

The results derived through this investigation revealed that forty days into the MCO, a substantial drop in the number of dengue cases occurred in Peninsular Malaysia. The dengue transmission process is complicating, and spatial variations need to be considered when it comes to issues relating to the host and vectors. Due to the restrictions imposed on travelling during the MCO, the scope of this investigation does not extend to semi-field evaluations on vector distributions. As such, it remains inconclusive at best, that the decrease in dengue cases is linked to the implementation of the MCO.

The factors leading to dengue transmission are multifaceted, and the spatial variation in host and vector contact rates are possibly the most important factors for DENV dynamics [24]. While the MCO enforced in Malaysia due to the COVID-19 pandemic, the impact of the host's large-scale movement restriction on the incidence of dengue can be analyzed. The trend was evaluated by comparing the major differences between the phases before and during MCO implementation with those of the same periods for previous years and simulation. It showed that COVID-19's MCO significantly influenced Malaysia's daily and weekly dengue incidence trend. These study results provide clear evidence from the analysis and complement the Reiner Jr et al. [22] and Falcón-Lezama et al. [5] studies which highlighting that the movement of people influenced dengue transmission using the simulation model. The early reduction in the incidence of dengue has also been recorded in India, and dengue cases have dropped by 50% over previous years. The declining incidences may have arisen at the beginning of MCO for several reasons: (1) With less outdoor hosts and therefore reduced vector-host interaction, as *Ae. aegypti* and *Ae. albopictus* are exophilic [21]. (2) the environmental alteration and comparatively lower artificial of vector breeding site due to less solid human waste; and (3) the reduced movement due to COVID-19 quarantine and home surveillance order (HSO) of infected patients.

Evidently, our research has shown that the dengue incidence rebounded earlier and increased at higher rate than in previous years, which demonstrated that the wide scale of the population's MCO approach in controlling the transmission of dengue is not viable. The result is consistent with the situation in Singapore, which had the most extreme dengue outbreak in seven years, and Jindal and Rao's [10] agent-based simulation model study showing significantly higher risk and severity of dengue transmission after COVID-19 MCO. The stay-at-home policy enable the host active most of the time in the indoor environment, increasing the endophagic *Ae. aegypti* biting tendencies to spread the virus. In contrast with Harrington et al. [7], who claimed that human transfer the virus aggressively between and within rural areas rather than mosquitoes due to restricting female *Ae. aegypti* flight range. The variation in Aedes larval population and high densities of biting mosquitoes in various month was related to the rainfall factors [8]. Rainfall always provides breeding habitat for mosquito as rainfall produce surface pools accumulated, and accompanying expansion of oviposition site, resulting a sustain population of mosquitoes. The extension of dry season or increment of temperature may result a reduction of adult mosquito. Thus, pattern on dengue transmission is influence by the abundance survival and behavioral of principle mosquitoes’ vector, the level of immunity to circulating virus in the local human population, density, distribution and movement of human *n* time required for development of virus in adult aedes mosquitoes. The abundance of vector population is pre-requisite for epidemic and transmission of dengue virus.

## 4 Conclusions

With this undertaking, we delved into the impact of the MCO on the dengue situation in Peninsular Malaysia. According to the results attained, the number of new dengue cases declined during the early stage of the MCO, but started to increase during the latter stages of the MCO. This information may prove to be useful to decision makers, during their efforts to formulate effective measures, for curbing the spread of dengue during the ongoing COVID-19 pandemic. It is worth mentioning that in the context of Malaysia, the infection and transmission rate of dengue surpasses that of COVID-19. It is our recommendation, that future work in this area include investigations on environmental parameters, and entomological as well as epidemiological issues.

## Declaration of competing interest

None.
